# Dynamics of Solid‐Electrolyte Interphase Formation on Silicon Electrodes Revealed by Combinatorial Electrochemical Screening

**DOI:** 10.1002/anie.202207184

**Published:** 2022-07-13

**Authors:** Daniel Martín‐Yerga, David C. Milan, Xiangdong Xu, Julia Fernández‐Vidal, Laura Whalley, Alexander J. Cowan, Laurence J. Hardwick, Patrick R. Unwin

**Affiliations:** ^1^ Department of Chemistry University of Warwick Coventry CV4 7AL UK; ^2^ Stephenson Institute of Renewable Energy Department of Chemistry University of Liverpool Liverpool L69 7ZF UK; ^3^ The Faraday Institution Quad One Harwell Campus Didcot OX11 0RA UK

**Keywords:** Electrochemistry, Li-Ion Batteries, Scanning Probe Microscopy, Silicon, Solid Electrolyte Interphase

## Abstract

Revealing how formation protocols influence the properties of the solid‐electrolyte interphase (SEI) on Si electrodes is key to developing the next generation of Li‐ion batteries. SEI understanding is, however, limited by the low‐throughput nature of conventional characterisation techniques. Herein, correlative scanning electrochemical cell microscopy (SECCM) and shell‐isolated nanoparticles for enhanced Raman spectroscopy (SHINERS) are used for combinatorial screening of the SEI formation under a broad experimental space (20 sets of different conditions with several repeats). This novel approach reveals the heterogeneous nature and dynamics of the SEI electrochemical properties and chemical composition on Si electrodes, which evolve in a characteristic manner as a function of cycle number. Correlative SECCM/SHINERS has the potential to screen thousands of candidate experiments on a variety of battery materials to accelerate the optimization of SEI formation methods, a key bottleneck in battery manufacturing.

## Graphical abstract

Scanning electrochemical cell microscopy (SECCM) enables combinatorial electrochemical assessment of a broad experimental space for solid‐electrolyte interphase (SEI) formation on Si electrodes for Li‐ion batteries, which is correlated to the heterogeneous and dynamic SEI chemistry through co‐located shell‐isolated nanoparticles for enhanced Raman spectroscopy (SHINERS).

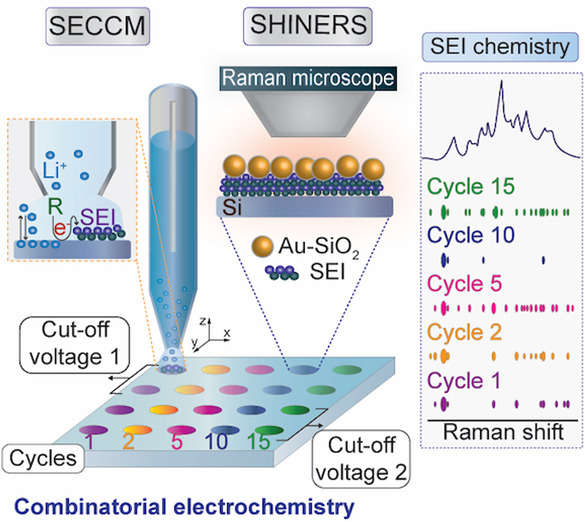

Li‐ion batteries^[1]^ are key for decarbonising energy and transportation systems. Silicon is promising as negative electrode in Li‐ion cells due to the higher theoretical specific capacity compared with graphite.[^2^, ^3^] However, Si undergoes large volume expansion during lithiation leading to instability of the solid‐electrolyte interphase (SEI)[^4^, ^5^] and mechanical failure.[^6^, ^7^] The SEI should ideally prevent continuous electrolyte decomposition, but cracking of the Si surface and the SEI breathing effect lead to a sustained loss of Li^+^ inventory.^[8]^ SEI composition and properties are affected by experimental formation conditions,^[9]^ but its characterisation is challenging as only a few techniques can provide meaningful chemical information. Nuclear magnetic resonance (NMR), Raman, Infrared, and X‐ray photoelectron spectroscopies (XPS) have been widely used to analyse the SEI composition.[^10^, ^11^] Raman spectroscopy can provide chemical information of SEI components^[12]^ but due to the low sensitivity,^[13]^ plasmonic amplification by Tip‐enhanced^[14]^ or Surface‐enhanced Raman spectroscopy (SERS)[^15^, ^16^] is usually required. A specific SERS approach is shell‐isolated nanoparticles for enhanced Raman spectroscopy (SHINERS) where Au‐SiO_2_ core–shell nanoparticles are used as plasmonic signal amplifiers. SHINERS provides access to many surface materials and morphologies,[^17^, ^18^, ^19^] and has been previously employed to study the SEI composition.[^20^, ^21^] A summary of state‐of‐the‐art studies using Raman‐based techniques for characterisation of SEI on Si electrodes is included in Table S1.

SEI understanding is, however, limited by the low‐throughput of conventional characterisation approaches, which make it impractical to study a large experimental space. Developing combinatorial strategies for high‐throughput exploration of SEI formation and properties is thus essential to accelerate the discovery of optimal SEI formation protocols, which is a key bottleneck in manufacturing of Li‐ion batteries. Combinatorial electrochemistry^[22]^ has been useful for screening electrocatalytic materials,[^23^, ^24^] but has been restricted to the use of complex multi‐channel cells in battery research.[^25^, ^26^, ^27^] While this strategy is promising to evaluate a library of materials, it is inadequate as a means at exploring a large experimental space. Consequently, efficient combinatorial electrochemical methods to rapidly screen experimental space in batteries are still required, with a clear potential to accelerate research timescales and reduce costs in battery development. In this regard, scanning electrochemical cell microscopy (SECCM)[^28^, ^29^] is a high‐throughput technique^[30]^ with high spatial resolution that allows thousands of individual electrochemical measurements to be made with control of experimental conditions. SECCM has been used to study positive electrode materials such as single LiMn_2_O_4_,^[31]^ and LiFePO_4_ ^[32]^ particles in aqueous electrolytes, but has only recently been implemented in a glovebox to study materials for Li‐ion cells under inert atmosphere,[^33^, ^34^] such as SEI formation on graphite.^[34]^

In this work, we report a powerful combinatorial screening method to characterise battery materials and its application to study SEI formation and properties on Si negative electrodes for Li‐ion cells. This combinatorial method is made possible by the high‐throughput spatially‐resolved nature of SECCM and correlative chemical analysis of the SEI composition by SHINERS. By screening a broad experimental space, we reveal the dynamics of the SEI formation on Si electrodes under different conditions. This novel combinatorial approach is widely applicable to other interfacial processes that control performance in battery materials beyond the SEI with the potential to screen thousands of candidate experimental conditions in a short time, which opens new avenues to significantly accelerate experimental research in battery materials.

The combinatorial correlative approach is illustrated in Figure 1, with full experimental details in the Supporting Information. SECCM (Figure 1A) uses a pipet probe (Figure S1), containing electrolyte solution and a quasi‐reference counter electrode (QRCE), to record local cyclic voltammetry (CV) measurements within the confined area defined by the liquid meniscus formed between the pipet and the surface of a monocrystalline Si wafer electrode with a (111) orientation. SEI formation is explored combinatorically by automated positioning of the pipet across the Si electrode at a series of predefined locations. An experimental space involving combinations of 2 different cut‐off voltages (+0.05 V and −0.13 V vs. Li/Li^+^, Figure S2), 5 different cycles of charge/discharge (1, 2, 5, 10 and 15 cycles) and 2 different electrolytes (1 M LiPF_6_ in propylene carbonate (PC) or ethylene carbonate/ethyl methyl carbonate (EC/EMC)) was studied (Figure S3). The cut‐off voltages were chosen to explore two state‐of‐charge (SOC) conditions: at low SOC (+0.05 V) where the SEI should be formed without severe mechanical changes on Si, and at high SOC (−0.13 V) where an overpotential below 0 V is reached, which can occur when operating under fast charging conditions.^[35]^ This combinatorial approach led to the SEI formation under 20 sets of different experimental conditions, with each set repeated for 11 or 13 times to collect significant statistics. The total number of individual electrochemical experiments was 244 taking only ca. 2 h of actual measurements and only covering a few hundred μm of the Si surface (Figure 1B shows SECCM footprints, with higher resolution in Figure S4). After combinatorial electrochemistry, SEI composition was analysed through co‐located SHINERS (Figure 1C) by placing Au‐SiO_2_ shell‐isolated nanoparticles (SHINs) (synthesis and characterisation details in Supporting Information, Figures S5–S9) on top of the already formed SEI on the Si surface.

**Figure 1:**
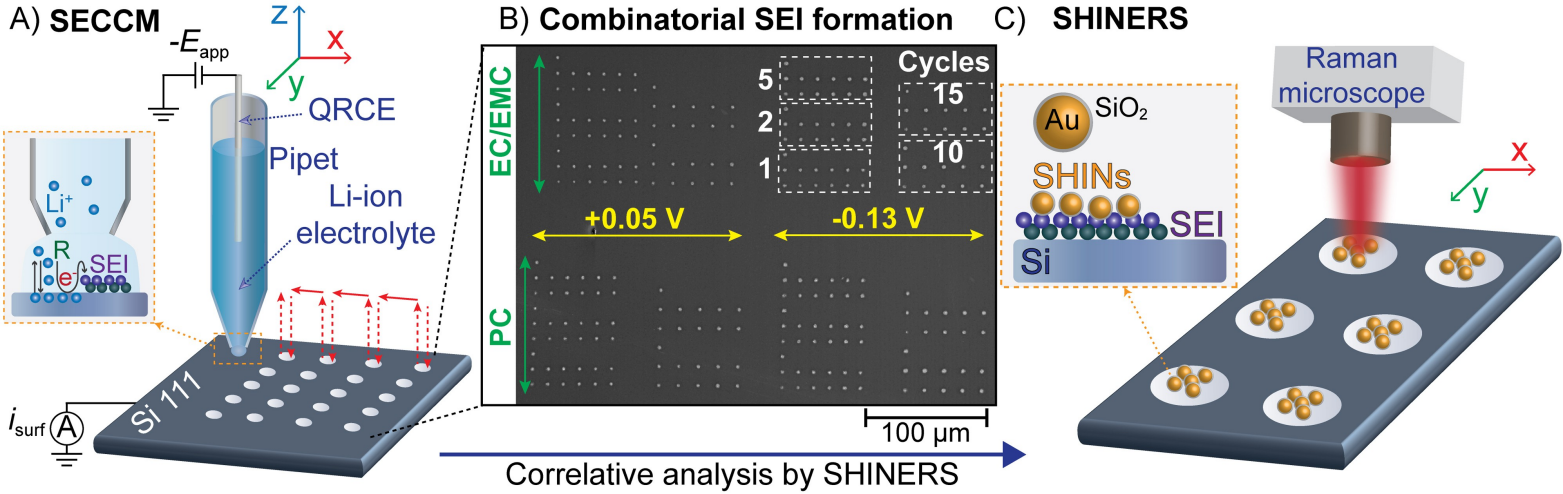
A) Schematic of automated SECCM for combinatorial electrochemical screening of the SEI formation. B) SECCM footprints imaged by scanning electron microscopy (SEM) after screening the SEI formation experimental space. C) Schematic of correlative chemical analysis of the SEI through SHINERS and Raman microscopy.

The first set of combinatorial experiments was performed in 1 M LiPF_6_ in PC. Figure 2 depicts average CVs for 1, 2, 5 and 15 charge/discharge cycles for the two SOC conditions. Small standard deviations indicate a homogeneous response across the Si surface. Two main cathodic processes (C1, C2) observed at low SOC (Figure 2A) are associated to electrolyte reduction and lithiation of the Si electrode.[^36^, ^37^] Cathodic current densities decreased upon cycling (see Figure S10 for full sequence) in agreement with the formation of the passivating SEI layer.^[38]^ A delithiation process (A1) located at a peak potential (*E*_pa_) of ca. +0.50 V is assigned to the phase transfer from Li_*x*_Si_*y*_ to amorphous Si. Delithiation current densities slightly increased upon cycling (Figure S11A) due to the generation of more accessible amorphous Si,^[6]^ whereas *E*_pa_ was consistent over cycling (Figure S11B). The appearance of only one delithiation process reinforces the idea of working under low SOC, with only one Li_*x*_Si_y_ phase formed, in contrast to macroscale experiments where two delithiation processes are usually observed.[^37^, ^39^] Under high SOC conditions, a crossover between cathodic and anodic sweeps (Figure 2B), which is characteristic of nucleation phenomena, suggests that Li plating occurred on the Si surface in addition to the processes already discussed at low SOC. Li plating is an important degradation mechanism in Li‐ion batteries and will lead to fresh surfaces for further SEI formation.

**Figure 2:**
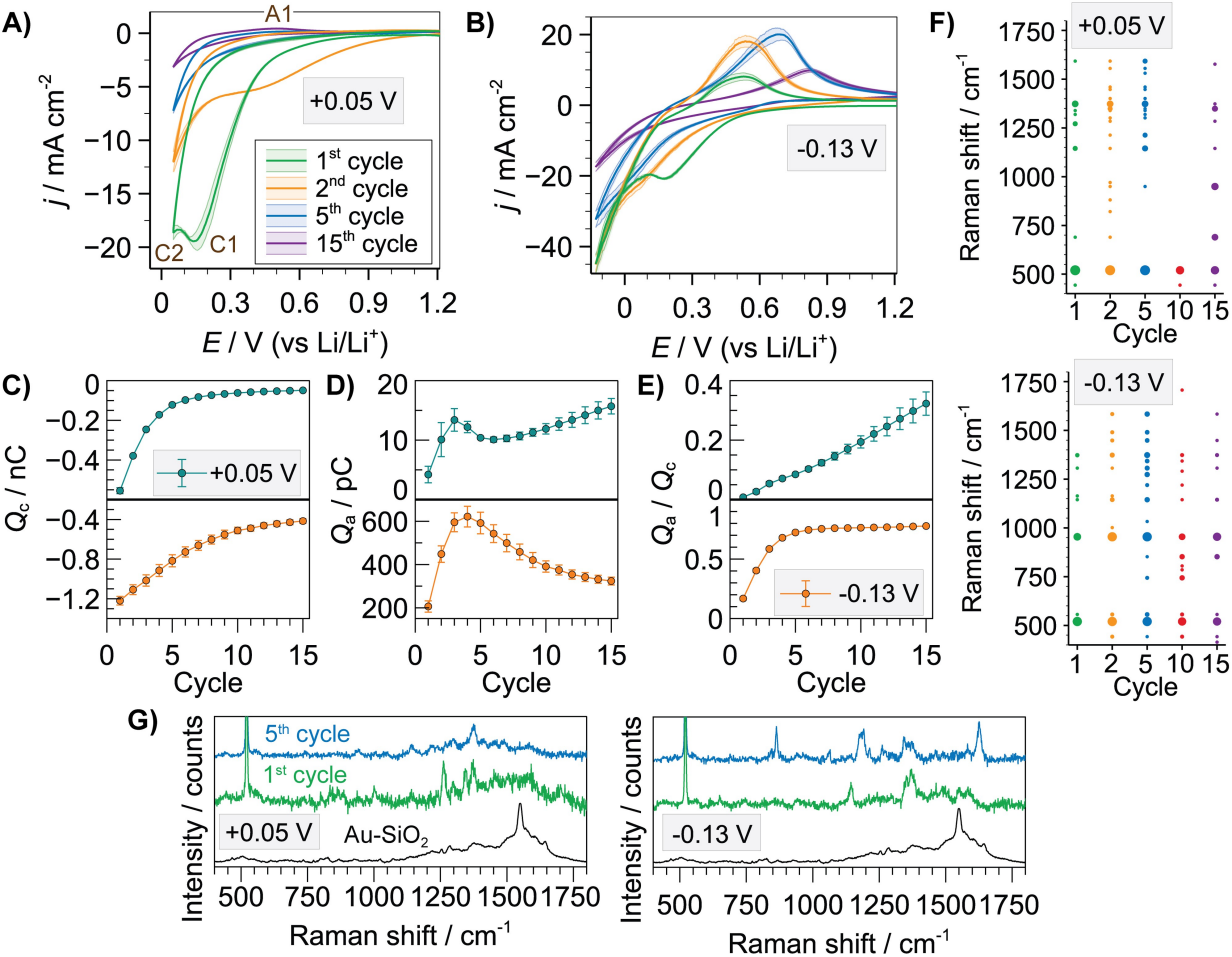
Averaged SECCM CVs (*n*=11) for 1, 2, 5 and 15 charge/discharge cycles in 1 M LiPF_6_ in PC with a cut‐off voltage of +0.05 V (A) or −0.13 V vs. Li/Li^+^ (B). Evolution of cathodic (*Q*_c_) (C), anodic (*Q*_a_) (D) charges and *Q*_a_/*Q*_c_ ratio (E) as a function of charge/discharge cycle. F) List of all Raman bands detected for the entire set of SECCM locations as a function of cycle number and SOC. Dot size represents the incidence number for a specific Raman band. G) Raman spectra for Au‐SiO_2_ SHINs and SEI formed upon 1 and 5 charge/discharge cycles under low and high SOC on Si wafer (note band at 520 cm^−1^ is from silicon wafer).

Cathodic current densities decreased upon cycling but the rate of decay in cathodic charge (*Q_c_*) (Figure 2C) was dependent on SOC conditions. Indeed, surface passivation (i.e. SEI formation) occurred largely over the first ≈5 cycles under low SOC but followed a more continuous, albeit slower growth under high SOC. Microscopic images of the SEI (Figures S12 and S13) show a general trend of increased thickness and coverage with cycle number, but with a rather large spatial heterogeneity (i.e. SEI is significantly rough). In both SOC cases, 15 cycles were not sufficient for a complete stabilisation in *Q_c_* as a result of the complex and dynamic nature of the SEI in the initial stages of formation. This fact is consistent with the SHINERS analysis where a diverse number of Raman bands between 400–1750 cm^−1^ were observed at different cycle number due to SEI formation (Figure 2F). Most differences were obtained between the 1^st^ and 5^th^ cycles, with the latter being particularly enhanced (Figure 2G), correlating with the electrochemistry. SEI components identified (see Table S2 for assignment of main Raman bands) were from a family of ROCO_2_Li and RCOOLi, as well as poly(ethylene) oxide (PEO) type species which agrees with previous SHINERS studies.^[20]^ Spectra are particularly rich in bands in the 1300–1500 cm^−1^ region that are assigned to C=O/CH_2_ modes. The main SEI forming reactions from the solvent are represented in Figure S14. Interestingly, a loss of Raman signal at 10 cycles was consistently detected and this correlated with increased background emission (Figure S15). High levels of emission have been previously assigned to LiPF_6_ decomposition.^[40]^ The decrease in intensity between cycles 1–5 demonstrates that emissive products have diffused away from the SEI and/or rate of decomposition has decreased or ceased due to SEI formation. The increase in emission at cycle 10 reinforces the fluctuating nature of the SEI, indicating increased accessibility of the LiPF_6_ to the electrode surface, this is in‐line with the Raman spectra, which shows fewer contributions from SEI products. After cycle 10 the emission again decreases indicating that SEI formation, as observed by Raman, correlates with decreased LiPF_6_ decomposition. This result signifies that the initial SEI formed on Si is not sufficiently passivating to prevent further reaction of LiPF_6_ to “P_*x*_F_*y*_O_*z*_” type species. Elemental mapping (Figure S16) underlines the lack of phosphorus species in the SEI, showing that the degraded salt species is highly mobile. SEI dynamic formation is associated to the SEI breathing effect,^[41]^ where species dissolve, re‐generate or evolve upon cycling, so it is reasonable that their relative concentrations or spatial arrangement might change throughout this process. This trend was observed for both SOC conditions, with only minor changes in the number and position of bands, which suggests that the main SEI chemistry is not significantly affected by the two different cut‐off voltages assessed herein.

The two SOC conditions also led to a different behaviour of the anodic charge (*Q*_a_) variation upon cycling (Figure 2D). *Q*_a_ generally increased upon cycling at low SOC whereas a significant decrease after a local maximum was observed at high SOC. The chemical interphase growing on Si under high SOC significantly hindered electron transfer kinetics as detected by a continuous shift in *E*_pa_ (Figure S17). Note that anodic processes could be a combination of delithiation and Li stripping under high SOC. *Q_a_/Q_c_* ratio (Figure 2E) provides certain information about charge/discharge efficiency of the Si electrode, with this ratio being strongly dominated by electrolyte reduction during the first cycles until the SEI passivates the Si surface. Both absolute values and trends upon cycling were different at low and high SOC, with low SOC showing a continuous increase whereas a sharp increase was observed at high SOC during the first cycles until reaching a plateau. However, the *Q_a_/Q_c_* was rather low at the 15^th^ cycle for both cases (0.32 and 0.78), suggesting that Li^+^ is still lost on side reactions as the SEI might be unable to provide full passivation,[^4^, ^37^] with formation of irreversible silicates^[42]^ and self‐discharge^[8]^ also possible.

The second set of combinatorial experiments was carried out using 1 M LiPF_6_ in EC/EMC. Voltammetric profiles (Figure 3A) were qualitatively similar to PC, in agreement with previous studies,^[37]^ suggesting that electrochemical reactions are not dominated by the solvent, but quantitatively higher current densities were measured for PC under low SOC. The first cathodic process had a peak potential of +0.23 V in EC/EMC (70 mV more negative in PC) whereas the delithiation process appeared at *E*_pa_ of +0.54 V in EC/EMC (40 mV more negative in PC), and was also relatively consistent upon cycling (Figure S18). There was a stronger effect of the solvent under high SOC conditions (Figure 3B). Current densities assigned to Li plating at the 1^st^ cycle were higher for EC/EMC, which is likely a consequence of higher coverage with passivating products in PC that hinders Li plating on the 1^st^ cycle. Anodic peak current densities were also higher in EC/EMC, but this behaviour changed after the 3^rd^ cycle, as a sharp decrease in current density took place in EC/EMC (see Figure S19 for full cycling sequence) and associated with a shift in *E*_pa_ (Figure S20).

**Figure 3:**
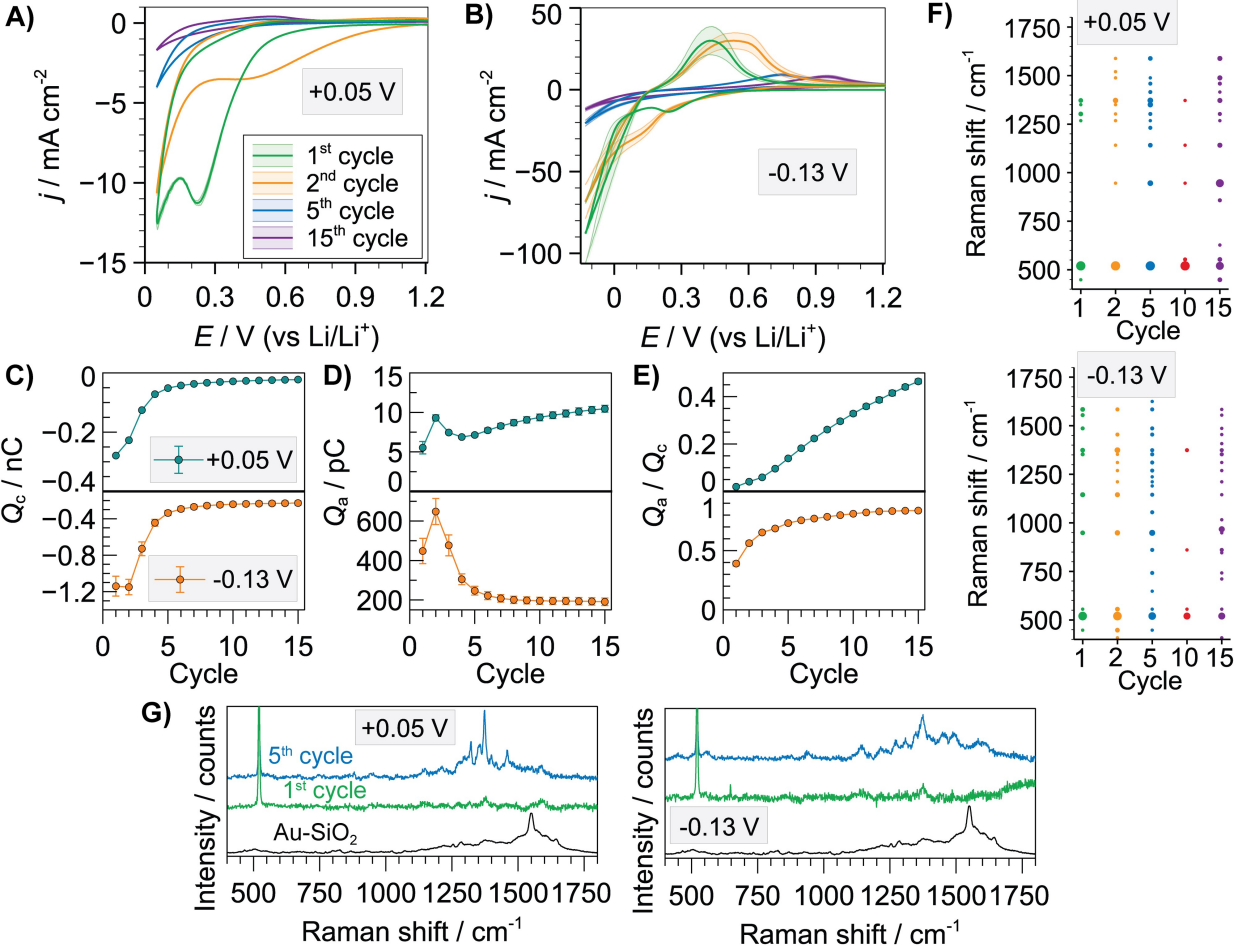
Averaged SECCM CVs (*n*=11) for 1, 2, 5 and 15 charge/discharge cycles in 1 M LiPF_6_ in EC/EMC with a cut‐off voltage of +0.05 V (A) or −0.13 V vs Li/Li^+^ (B). Evolution of cathodic (*Q*_c_) (C), anodic (*Q*_a_) (D) charges and *Q*_a_/*Q*_c_ ratio (E) as a function of charge/discharge cycle. F) List of all Raman bands detected for the entire set of SECCM locations as a function of cycle number and SOC. Dot size represents the incidence number for a specific Raman band. G) Raman spectra for Au‐SiO_2_ SHINs and SEI formed upon 1 and 5 charge/discharge cycles under low and high SOC (note band at 520 cm^−1^ is from silicon wafer).

The *Q*_c_ trend (Figure 3C) provides information on the SEI formation, with a similar trend upon cycling under the two SOC and most of the surface passivation taking place in the initial 4–5 cycles. Microscopic analysis of the SEI again showed a general increase in coverage upon cycling for low SOC, being relatively more constant for high SOC (after 2^nd^ cycle), but with a rough morphology and locally heterogeneous thickness (Figures S21 and S22). SHINERS analysis again reveals the SEI dynamic formation and evolution with EC/EMC, as a consistent set of SHINERS bands was not obtained for all charge/discharge cycles (Figure 3F). Increased intensity of Raman bands at the 5^th^ cycle compared to the 1^st^ cycle (Figure 3G) indicates the enrichment of SEI products commensurate with the observed electrochemical surface passivation. The emission background increases again for the 10^th^ cycle (Figure S23), which conceals some bands, with some recovery at the 15^th^ cycle. Main SEI components detected (see Table S2 for assignment of main Raman bands) under these conditions were lithium methylene carbonate (LMC), PEO and ROCO_2_Li and RCOOLi species (see reactions in Figure S14). The spectra are inconclusive on the specific formation of either lithium ethylene monocarbonate (LEMC) or lithium ethylene dicarbonate (LEDC) that have been previously observed on materials such as Sn.^[21]^ The moderate differences in number and position of SHINERS bands for the entire experimental space (Figure S24) probed by the correlative combinatorial approach highlight the dynamics of SEI formation, which are particularly controlled by cycling, with only a minor effect from the cut‐off voltages assessed herein. Significant SEI Raman bands would have been expected after cycle 1 due to the large electrolyte reduction wave ca. +0.2 V for all conditions. However, the spectroscopic data is limited, confirming that the initial SEI film is unstable and a considerable amount of SEI species goes into solution, which is reinforced by AFM and SEM data. The major spectral variations between PC and EC/EMC are in fact seen in the 1^st^ cycle due to differences in solubilities of initial reduction products, with EC/EMC products being more soluble as barely any bands are observable. After further cycling, the main species detected by SHINERS are polymeric (PEO‐type), which have lower solubility and measured differences between the SEIs from PC and EC/EMC are minor.

The *Q_a_* trend upon cycling (Figure 3D) was similar to that in PC, with a slightly steady increase under low SOC but a sharp decrease after a local maximum under high SOC. The trend in *Q*_a_/*Q*_c_ (Figure 3E) was also similar to that found in PC with relatively low absolute values (0.46 and 0.83 under low and high SOC, respectively), which again evidences an unstable Si interface also in EC/EMC that led to loss of Li^+^ on side reactions.

In summary, a powerful method to explore the combinatorial high‐throughput formation and analysis of the SEI on Si electrodes for Li‐ion cells under a broad experimental space (20 sets of conditions, with several repetitions to collect significant statistics) has been demonstrated. Through this novel correlative SECCM/SHINERS approach, the dynamics of the SEI formation on Si electrodes was revealed, which is shown to be particularly dependent on experimental variables such as cycle number. The Raman analysis supports the important observation that the SEI formed upon cycling on Si negative electrodes is continuously evolving coupled with instability towards the LiPF_6_ salt. This is an important distinction to SEIs formed on graphitic carbon whereby general formation protocols result in SEIs that have minimal parasitic current consumption after ca. 2 cycles. Our work highlights the major challenge to be overcome if electrodes containing a high fraction of Si are to be commercialised, whereby focused research in developing SEIs as stable as those found in graphite is required.

SHINERS were placed onto an already formed SEI, thereby the outer SEI layer (i.e. SEI/electrolyte interface), which is more polymeric in nature, is detected. Future work will investigate the combination of SECCM with SHINERS already positioned onto the electrode surface, where they would preferentially enhance the electrode/SEI interface, thereby accessing information on the inner inorganic and organic layers, with use of a Kerr Gate to suppress fluorescence effects.^[40]^

This combinatorial approach has the potential to provide huge datasets of correlated electrochemical and chemical information to facilitate the understanding of complex interfacial phenomena in battery materials. Screening thousands of candidate experimental conditions would drive the discovery of high‐performing ones, which could be later assessed by more conventional battery techniques. This development opens new avenues to significantly accelerate the experimental throughput in battery research being widely applicable to different materials.

## Conflict of interest

The authors declare no conflict of interest.

## Supporting information

As a service to our authors and readers, this journal provides supporting information supplied by the authors. Such materials are peer reviewed and may be re‐organized for online delivery, but are not copy‐edited or typeset. Technical support issues arising from supporting information (other than missing files) should be addressed to the authors.

Supporting Information

## Data Availability

The data that support the findings of this study are openly available in Zenodo^[43]^ at https://doi.org/10.5281/zenodo.6545399.
