# Supplementary material for: Dynamics of Solid‐Electrolyte Interphase Formation on Silicon Electrodes Revealed by Combinatorial Electrochemical Screening
Source: Angew Chem Int Ed Engl. 2022 Jul 13;61(34):e202207184. doi: 10.1002/anie.202207184 (PMC9543478; doi:10.1002/anie.202207184)
Supplement: Supplementary file 1 — Supporting Information [file ANIE-61-0-s001.pdf]

Angewandte Chemie
Eine Zeitschrift der Gesellschaft Deutscher Chemiker

Supporting Information

Dynamics of Solid-Electrolyte Interphase Formation on Silicon Electrodes Revealed by Combinatorial Electrochemical Screening

D. Martín-Yerga, D. C. Milan, X. Xu, J. Fernández-Vidal, L. Whalley, A. J. Cowan, L. J. Hardwick, P. R. Unwin**

S1. EXPERIMENTAL

Chemicals and materials for electrochemical experiments

Battery-grade lithium hexafluorophosphate solutions in ethylene carbonate (EC) and ethyl methyl carbonate (1.0 M $LiPF_6$ in EC/EMC=50/50 (v/v); Sigma-Aldrich) and propylene carbonate (1.0 M $LiPF_6$ in PC) were used as received. These solutions contained <15 ppm of H_2O and <50 ppm of HF according to the manufacturer.

A monocrystalline (111) Si wafer (1-10 Ω•cm, n-type, P-doped, 381±25 μm thick, single-side polished) was used as working electrode. The Si wafer was cleaved in small pieces (< 1 cm) but used as received without any chemical cleaning to keep the native oxide layer intact. The Si electrode was electrically connected by placing conductive copper tape on the back of the wafer.

Ag wires (0.25 mm diameter, 99.99%, Goodfellow) were used as quasi-reference counter electrodes (QRCE). Ag QRCEs have been previously used successfully for electrochemical microscopy experiments of Li-ion battery materials.[1,2] The Ag QRCE potential was converted to the Li/Li^+ scale after calibration against a Li electrode (ribbon, 99.9% purity, Sigma-Aldrich) under the working solution (1 M $LiPF_6$ in EC/EMC or PC).

Combinatorial scanning electrochemical cell microscopy (SECCM)

SECCM measurements were carried out using a home-built instrument installed in an Ar-filled glovebox (MBraun MB200B/MB20G) with levels of H_2O < 0.2 ppm and O_2 < 0.1 ppm, as previously reported.[2] Single-channel pipet probes were fabricated with a CO_2-laser puller (P2000, Sutter Instruments) using borosilicate filamented capillaries (1.2 mm outer diameter, 0.69 mm inner diameter, Harvard Apparatus).

Pipet probes had an opening diameter of ca. 1 μm as measured by a scanning electron microscopy (SEM) on a ZEISS Gemini microscope (Figure S1). Before the experiment, they were filled with Li-ion electrolyte solution using a MicroFil syringe (World Precision Instruments). Automated translation of the pipet probe was carried out with a 3-axis xyz piezoelectric positioner (P-611.3S NanoCube, Physik Instruments).

An SECCM measurement involved approaching the pipet probe to the Si electrode surface, during which a voltage ($-E_{app}$) of +1.25 V (vs Li/Li^+) was applied between the Ag QRCE inserted into the pipet and the Si electrode. The measured surface current (i_{surf}) was used as a feedback signal to detect when the liquid meniscus at the tip of the pipet contacted the Si surface. When a threshold of 2.0 pA was attained, the pipet movement was stopped and a cyclic voltammetry (CV) was recorded within the confined area defined by the meniscus (droplet cell) formed between the probe tip and the Si surface. Note that the pipet did not physically contact the electrode surface. The voltammetric sweep was carried out between $-E_{app}$ and a cut-off voltage (vertex) at a scan rate of 1 V s^{-1} in order to examine rapid phenomena in the early stages of the solid-electrolyte interphase (SEI) formation. The i_{surf} was recorded by a custom-built electrometer with a data acquisition rate of 2.57 ms, where the current was sampled every 10 μs and averaged 256 times with one extra iteration for transferring data to the computer. This measurement protocol was repeated at a series

of predefined locations on the Si surface through an automated hopping regime consisting of pipet approach to the surface at a rate of 2 μm s^{-1}, electrochemical measurement, retract at 2 μm s^{-1}, and x-y translation at 2 μm s^{-1}, as previously reported.[3,4] The lateral separation between each individual measurement was set to 12 or 15 μm to prevent spatial overlap of the probed areas.

SECCM was deployed under a combinatorial protocol to evaluate the SEI formation under a broad experimental space by translating the pipet probe to another location and repeating the measurement protocol under a different set of conditions. This approach involved the combination of two different cut-off voltages (+0.05 V and -0.13 V vs Li/Li^+, Figure S2) and a different number of charge/discharge cycles (1, 2, 5, 10, 15 cycles). The same protocol was repeated using a new pipet probe filled with a different electrolyte (1 M $LiPF_6$ in EC/EMC or 1 M $LiPF_6$ in PC). Full combinatorial space is illustrated in Figure S3 and the footprint left by SECCM measurements is shown in Figure S4. Current densities are given normalised by the geometric area of the SECCM footprints. Note that to facilitate the finding of SECCM footprints and avoid any possible dissolution of SEI components and compositional changes, the Si samples were not rinsed after SECCM to carry out the SHINERS analysis. As a consequence, they contain a small amount of residual electrolyte salts. Samples were kept under inert atmosphere during transfer between instruments.

The SECCM setup (sample and positioner) was placed on a bench-top vibration isolation platform (BM-10, Minus K Technology) to minimize mechanical vibrations and covered with a copper woven mesh (60 mesh per inch, 0.166 mm wire diameter, Cadisch Precision Meshes) acting as Faraday cage to reduce electrical noise. Data acquisition and instrumental control in SECCM was achieved using a FPGA card (PCIe-7852R) controlled by a LabVIEW 2020 (National Instruments) interface running the Warwick electrochemical scanning probe microscopy (WEC-SPM, www.warwick.ac.uk/electrochemistry) software. Data processing and analysis was carried out with a code written in-house in Python language with the aid of SciPy libraries.[5]

Microscopic characterisation of the SEI after SECCM

The morphology and thickness of the SEI was analysed by atomic force microscopy (AFM) and SEM, whereas elemental mapping was recorded by energy-dispersive X-ray spectroscopy (EDX). AFM was carried out using an Innova microscope (Bruker) in tapping mode with antimony (n) doped Si probes (RFESP-75, Bruker). Scans were recorded with 256 points per line at 0.1 Hz. AFM images were analysed with the Gwyddion software (Czech Metrology Institute). Note that for the microscopic characterisation of the SEI, the electrolyte residues from the SECCM experiment were removed by gently dipping the Si sample in dimethyl carbonate (DMC) for a few seconds. While this is necessary to characterise the actual thickness and morphology of the SEI, without any interference from electrolyte residues, we acknowledge that some soluble SEI components might be dissolved through this process (*vide infra*); our goal was to obtain semi-quantitative insight and trends of SEI thickness as a function of cycle number.

Synthesis of gold nanoparticles

Citrate-stabilised gold nanoparticles (Au NPs) were obtained by the citrate reduction method described by Turkevich and Frens[6–9] where 200 mL of a 0.01 % $HAuCl_4$ (99.995 %, Sigma-Aldrich) solution was left to boil under strong stirring. 1.5 mL of 1 % citric acid (≥ 99.5 %, Sigma-Aldrich) were immediately added to the solution of $HAuCl_4$ and the solution changed from pale yellow to maroon after a few minutes. The dispersion of nanoparticles was then allowed to cool under stirring and the resultant suspension was stored away from light sources for the duration of the experiments, remaining stable throughout the process.

Synthesis of SiO_2-coated shell isolated nanoparticles (SHINs)

SiO_2-coatings for SHINs were obtained following the protocol described by Tian et al.[10] 15 mL of gold nanoparticles (~0.05 nM) were placed in a round bottomed flask under strong stirring. 200 µL of a 1 mM, freshly prepared (3-Aminopropyl)-trimethoxysilane (APTMS) (97%, Alfa Aesar) solution were added dropwise and left under vigorous stirring for 20 minutes at room temperature. 1.6 mL of a 0.54% fresh sodium silicate solution (Na_2SiO_3) (≥10% NaOH, ≥10% SiO_2 basis, Sigma-Aldrich) were added dropwise and allowed to stir for another 3 minutes before transferring the flask to a hot water bath at 98°C. Suitable, pinhole-free SHINs were obtained after 30 minutes reaction time.

Characterisation of Au NPs and SHINs

Au NPs were characterised by UV-Visible absorption spectroscopy (Figure S5). The position of the absorption band was used to determine the average size of the Au NPs. Au NPs had an estimated diameter of 68 nm, as determined by the method proposed by Haiss[11] and co-workers, for particles in the range of 35-110 nm diameter.

Differential centrifugal sedimentation (DCS) was carried out to investigate the SiO_2 coating process of AuNPs (Figure S6). Au NPs had a diameter of $57 \pm 9.2 \times 10^{-3}$ nm according to DCS with a resolution of ±0.1 nm.[12] SiO_2 shell thicknesses of 2, 4 and 8 nm were estimated for 15, 30 and 60 minutes respectively, following the iterative fitting described by the literature.[12,13] A disc centrifuge (DC24000, CPS Instruments Inc.) operated at 24000 rpm was used for DCS. Spin gradient was created by progressive addition of 8 and 24 wt % fresh sucrose (≥ 99.5%, Sigma-Aldrich) solutions. Calibration of the equipment was achieved before each measurement with polyvinyl chloride (PVC) particles (~0.263 µm, Analytik Ltd) as a standard. All measurements were performed at least three times to ensure the reproducibility of the data.

Pinhole and enhancement tests were performed to ensure the suitability of the SiO_2-coated Au NPs for SHINERS.[14] No pinholes were observed for SHINs deposited on Si wafer while a large enhancement was observed for SHINs deposited onto Au wafer (Figure S7). These tests were carried out with a Raman microscope (Renishaw InVia) using a 633 nm laser at 10 %. Si wafer was used to calibrate the Raman with a resolution of 1.1 cm^{-1}. For Raman pinhole tests, 5 µL of concentrated SiO_2-coated SHINs (~ 1.13 nM) were deposited onto a silicon wafer (Si (100), Agar Scientific) and 2 µL of a 10 mM pyridine (99.8 %,

Sigma-Aldrich) solution were added on top of the SHINs. Enhancement tests were similarly performed using a gold wafer (Au (111), Platypus Technologies).

Co-located Shell-isolated nanoparticle-enhanced Raman spectroscopy (SHINERS)

Co-located SHINERS was performed on Si samples following combinatorial SECCM to analyse the chemical composition of the SEI layer. SHINs were dispersed in MilliQ water, and a drop deposited on a glass window. After drying in vacuum at 100 °C (overnight), the glass window with dispersed SHINs was placed in close contact with the SECCM Si sample on top of the already formed SEI (Figure S8). These *ex situ* cells were assembled under argon atmosphere in glovebox with 0.1 ppm H_2O and O_2 levels.

A Raman microscope (Renishaw InVia) with a 633 nm laser as excitation source (power < 300 μW) was then used to record the Raman measurements. The laser was focused onto individual SECCM spots with a 50x objective. Raman spectra were collected with 5 accumulations and 30 s exposure time for each SECCM spot. Conditions were optimized to ensure good signal-to-noise ratio to resolve Raman bands. Raman spectra were baseline-corrected using the baseline correction tool of the WiRE 4.0 software (Renishaw). A series of Raman spectra recorded for a set of SECCM repeats under the same conditions are shown in Figure S9. The spectra are variable depending on spot, which could be a result of SHINs density, heterogeneous enhancement and/or heterogeneous nature of SEI.

S2. ADDITIONAL FIGURES AND TABLES

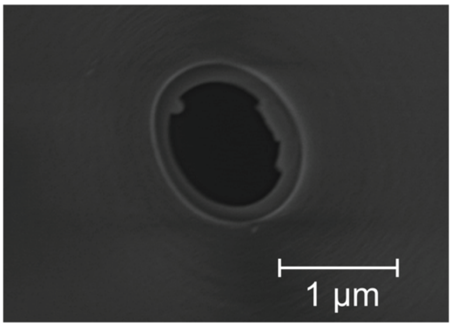

Figure S1. SEM image of a typical single-channel pipet probe used for the SECCM measurements. The tip diameter was ca. 1 μm. Images were taken at an acceleration voltage of 5 kV using the InLens detector.

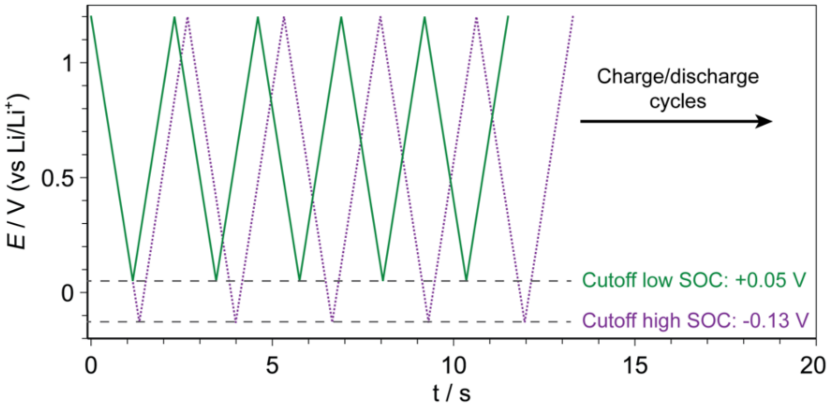

Figure S2. Schematic illustrating the two voltammetric programmes applied by SECCM to evaluate the SEI formation under different conditions of state-of-charge (SOC). Cut-off voltages of +0.05 V and -0.13 V (vs Li/Li^+) were selected for low and high SOC conditions, respectively.

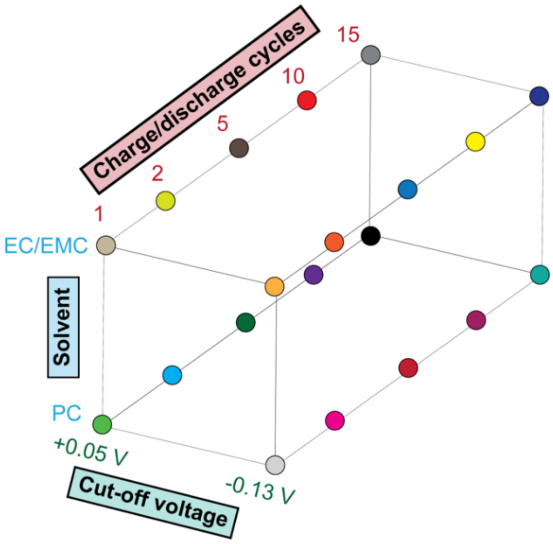

Figure S3. Schematic of the combinatorial experimental space probed by SECCM consisting of two different solvents (PC, EC/EMC), two different cut-off voltages (+0.05 V, -0.13 V) and 5 different charge/discharge cycles (1, 2, 5, 10, 15). 11 or 13 repetitions of each set of conditions was repeated to collect significant statistics.

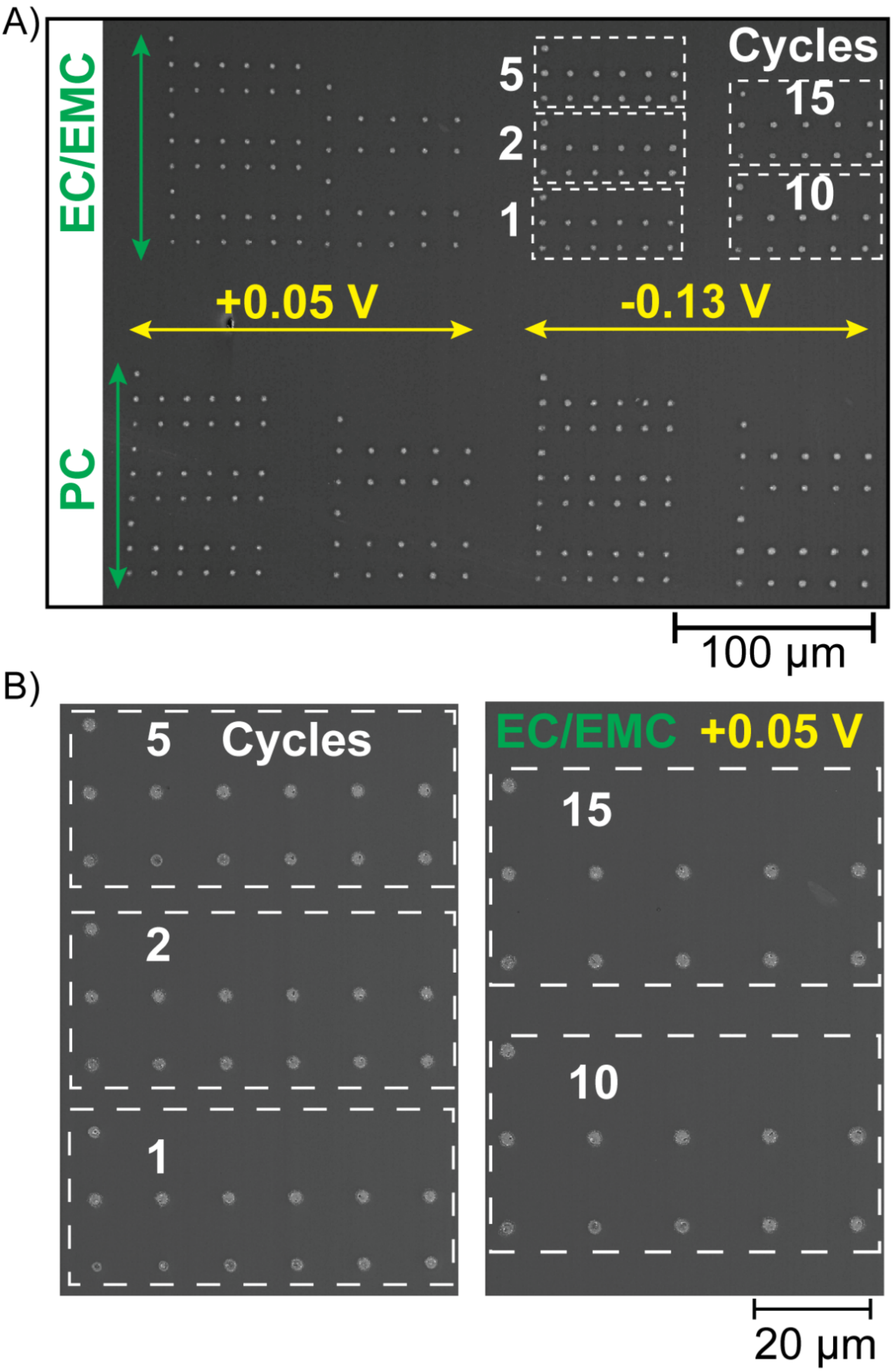

Figure S4. (A) SEM image of the SECCM footprint left after screening a large experimental space consisting of two Li-ion battery electrolytes (1 M $LiPF_6$ in EC/EMC and PC), two cut-off voltages (+0.05 and -0.13 V vs Li/Li^+) and five different charge/discharge cycles (1, 2, 5, 10, 15). **(B)** Higher magnification of the SECCM footprint for the experiments recorded with 1 M $LiPF_6$ in EC/EMC under a cut-off voltage of +0.05 V (vs Li/Li^+) for 1, 2, 5, 10 and 15 charge/discharge cycles. Images were taken at an acceleration voltage of 3 kV using the InLens detector.

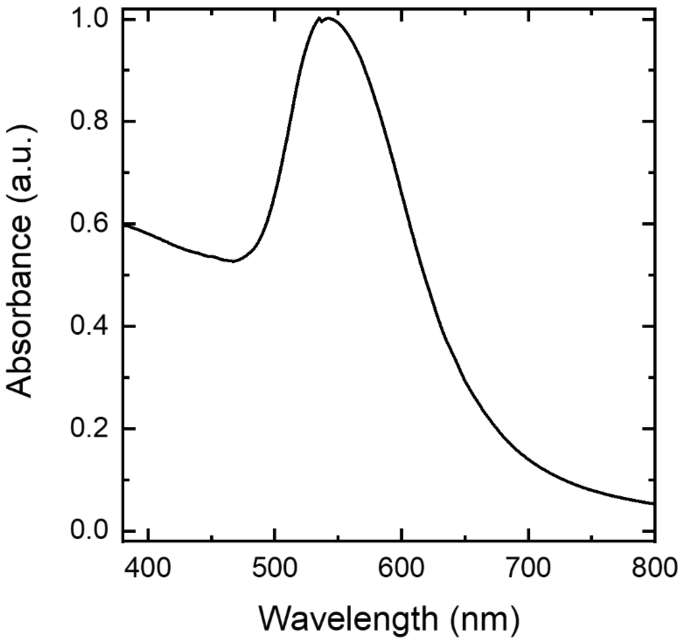

Figure S5. Normalised UV-Visible absorption spectrum of Au NPs used as cores for SHINERS.

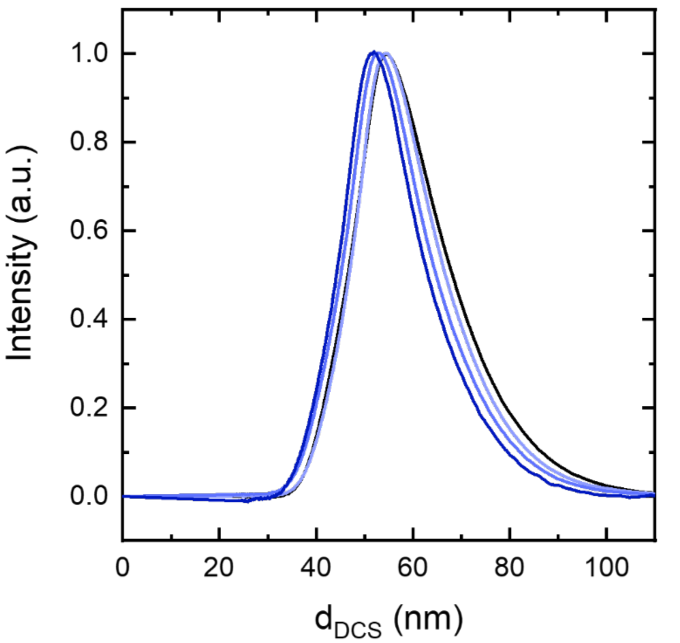

Figure S6. Normalised Differential Centrifugal Sedimentation (DCS) spectra of citrate-stabilised Au NPs **(-)** and SiO_2-coated SHINERS after 15 (-), 30 (-), and 60 (-) min of reaction time. Au NPs had a diameter of 57 with a standard deviation of $9.2x10^{-3}$ nm according to DCS with a resolution of ±0.1 nm.[12] Shell thicknesses of 2, 4 and 8 nm were estimated for 15, 30 and 60 minutes respectively, following the iterative fitting described by the literature.[12,13]

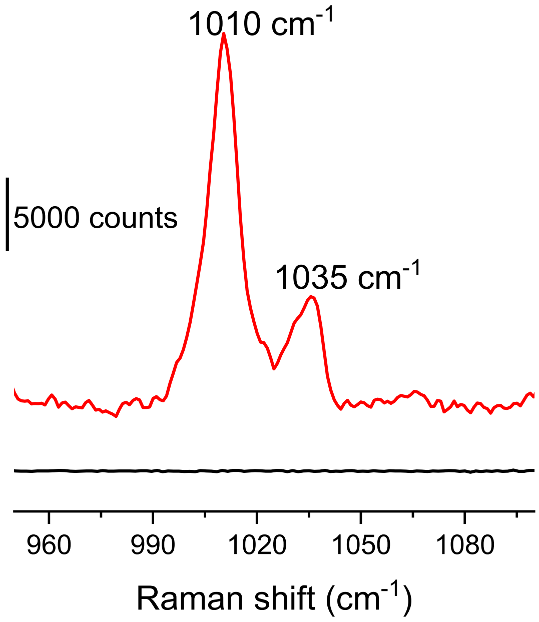

Figure S7. Raman spectra obtained for pinhole and enhancement tests of the SHINs in contact with pyridine. No pinholes were observed for SHINs deposited on Si wafer **(-)** while a large enhancement is observed for SHINs deposited onto Au wafer **(-)**.

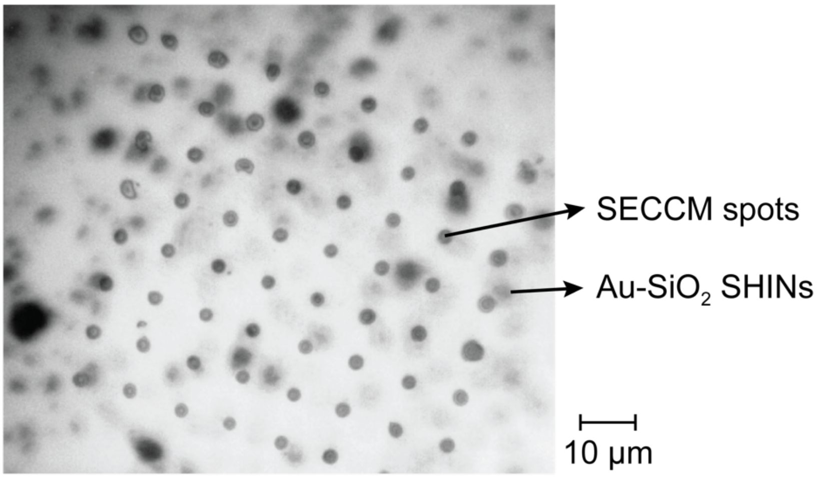

Figure S8. Optical microscopy image showing Au-SiO_2 SHINs placed in contact with the SECCM Si sample on top of already formed SEI spots. Note that this array pattern is slightly different to that actually used for the full combinatorial experiment (shown in Figure S4).

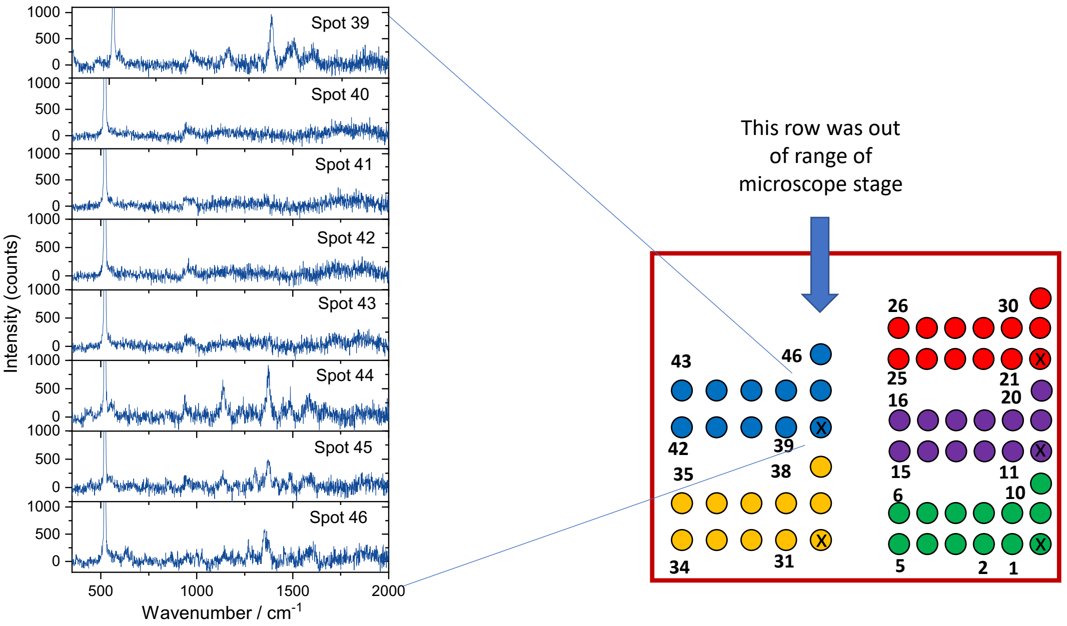

Figure S9. SHINERS spectra series recorded for 1 M $LiPF_6$ in PC with a voltage cut-off of +0.05 V vs. Li^+/Li for 15 charge/discharge cycles. 8 SECCM spots were measured that were within microscope stage mapping range. The full Raman data set for all conditions is available at DOI: 10.5281/zenodo.6545400

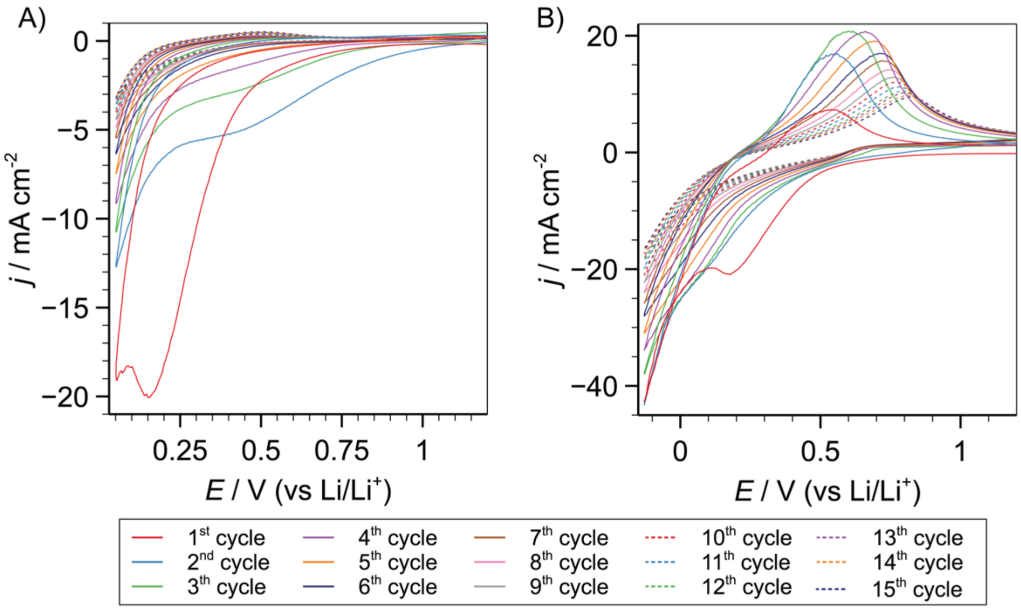

Figure S10. Representative CVs for 15 consecutive cycles recorded by SECCM in 1 M $LiPF_6$ in PC under low **(A)** and high **(B)** SOC (cut-off voltages of +0.05 and -0.13 V vs Li/Li^+, respectively).

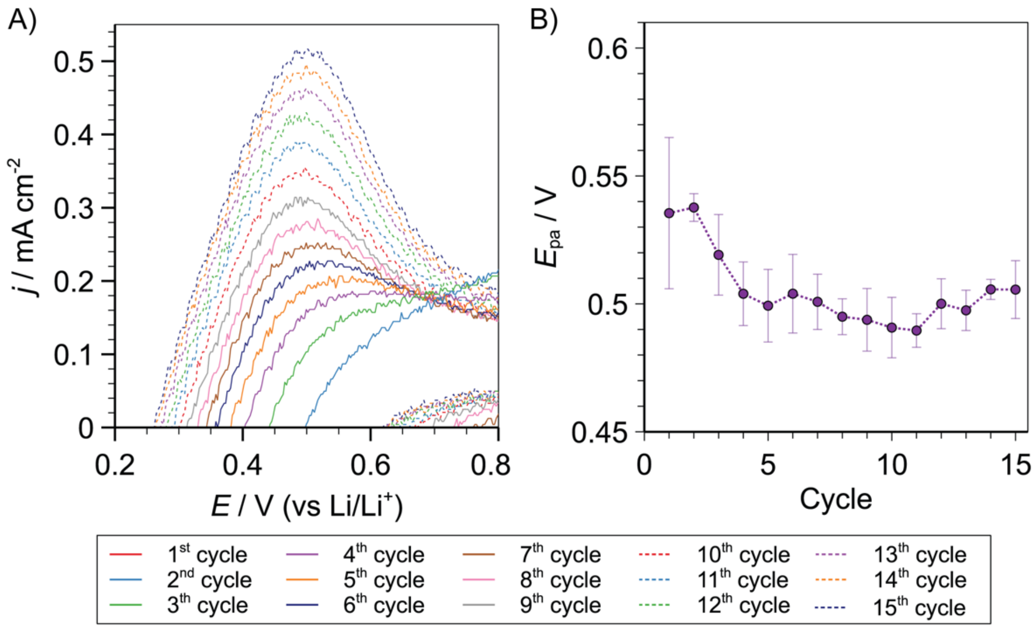

Figure S11. (A) Anodic component of representative CVs for 15 consecutive cycles recorded by SECCM in 1 M $LiPF_6$ in PC under low SOC (cut-off voltage: +0.05 V vs Li/Li^+). **(B)** Evolution of the anodic peak potential (E_{pa}) in function of charge/discharge cycle. E_{pa} values are the average of all the SECCM individual measurements (n=11).

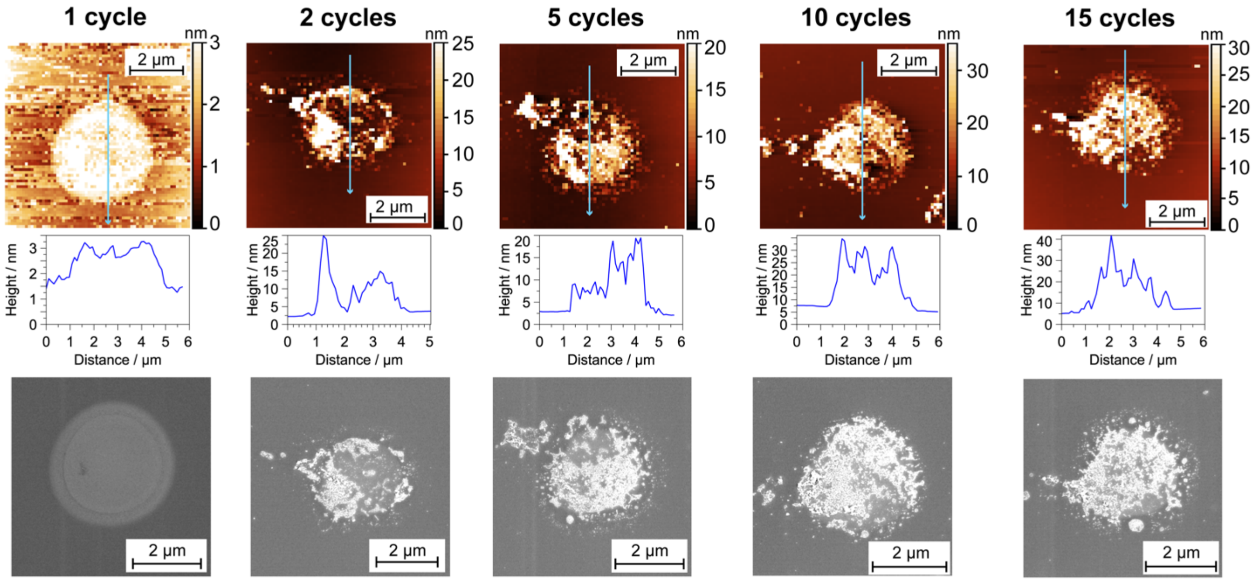

Figure S12. AFM topography images with corresponding height profiles and SEM images for the SEI layer prepared through SECCM for 1, 2, 5, 10 and 15 cycles in 1 M $LiPF_6$ in PC under low SOC (cut-off voltage: +0.05 V vs Li/Li^+). Arrows indicate the location and direction where the height profiles were extracted. Note that some soluble components might be dissolved when removing the electrolyte residue with DMC, which might explain the particular characteristics of the SEI at cycle 1 (very thin but homogeneous layer) compared to all other cases.

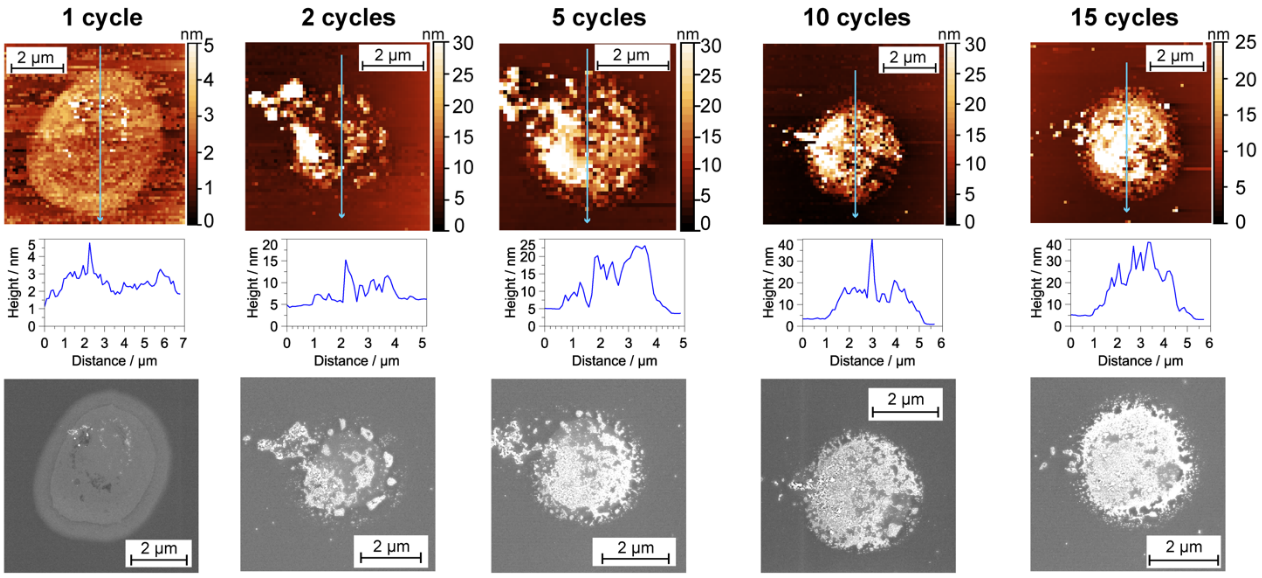

Figure S13. AFM topography images with corresponding height profiles and SEM images for the SEI layer prepared through SECCM for 1, 2, 5, 10 and 15 cycles in 1 M $LiPF_6$ in PC under high SOC (cut-off voltage: -0.13 V vs Li/Li^+). Arrows indicate the location and direction where the height profiles were extracted.

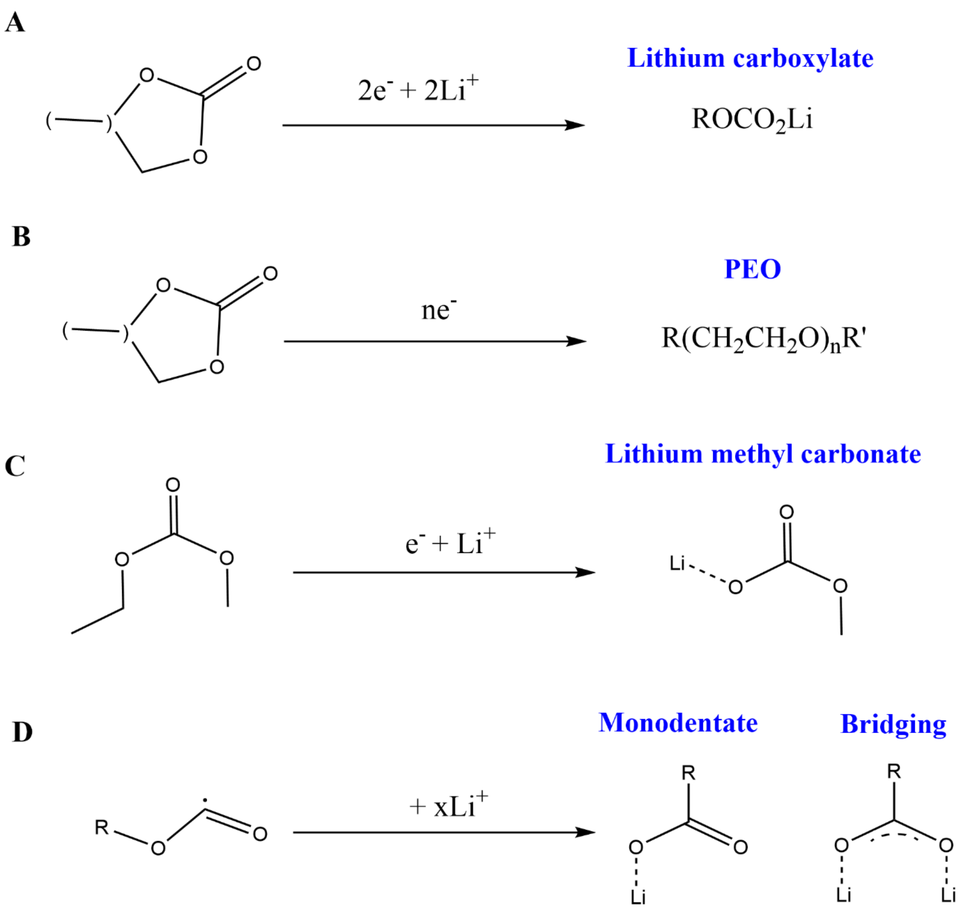

Figure S14. Schematic of main SEI forming processes from solvent reactions, derived from Raman measurements. Expected gases released from the reduction reactions have been omitted as they have not been confirmed from this study.

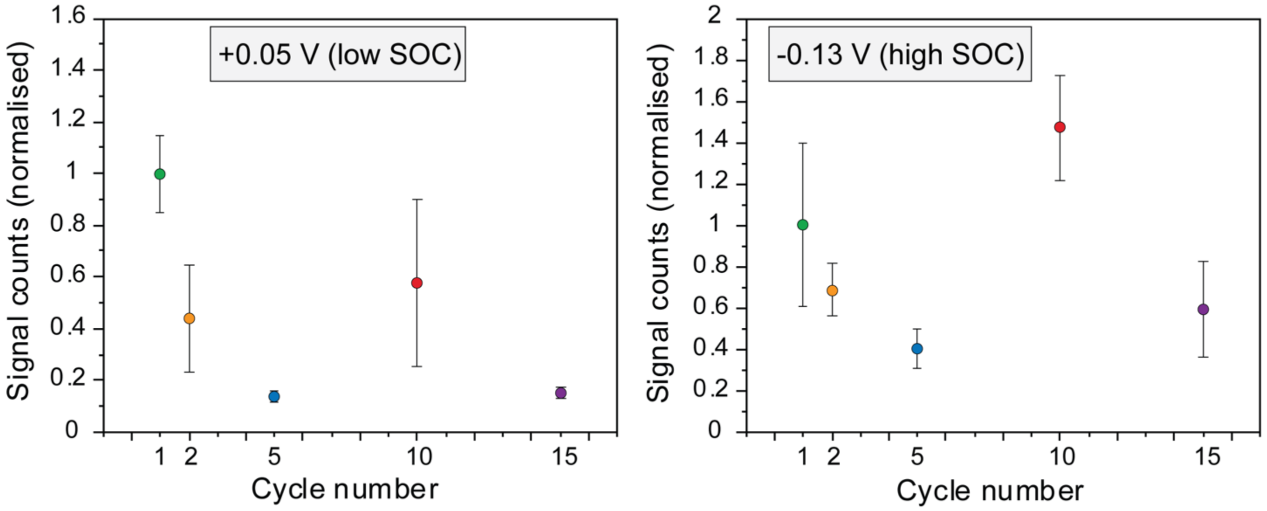

Figure S15. Averaged background emission intensity (normalised to the intensity of cycle 1) prior to baseline correction across all detected Raman bands in function of cycle number for the entire set of SECCM locations in 1 M $LiPF_6$ in PC at low SOC (cut-off voltage: +0.05 V vs Li/Li^+) and high SOC (cut-off voltage: -0.13 V vs Li/Li^+). Error bars represent the standard deviation at each wavelength of identified bands recorded for all different SECCM locations.

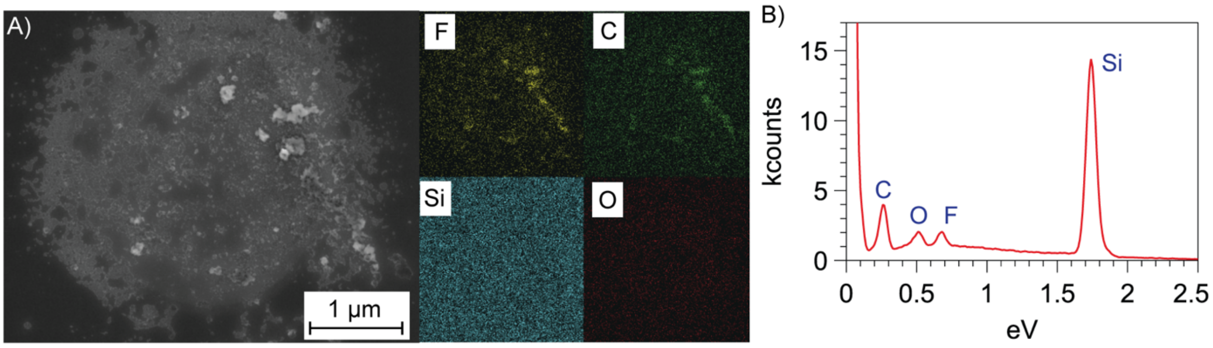

Figure S16. (a) SEM image and EDX mapping with (b) corresponding spectrum recorded for the SEI layer at a specific SECCM spot. This analysis shows increased concentration of C-based and F-based species on the SEI. P was not detected that suggests that LiF are the main F-based species and also discards the presence of rests of electrolyte residue ($LiPF_6$). SEI was formed through 15 charge/discharge cycles in 1 M $LiPF_6$ in PC under low SOC (cut-off voltage: +0.05 V vs Li/Li^+).

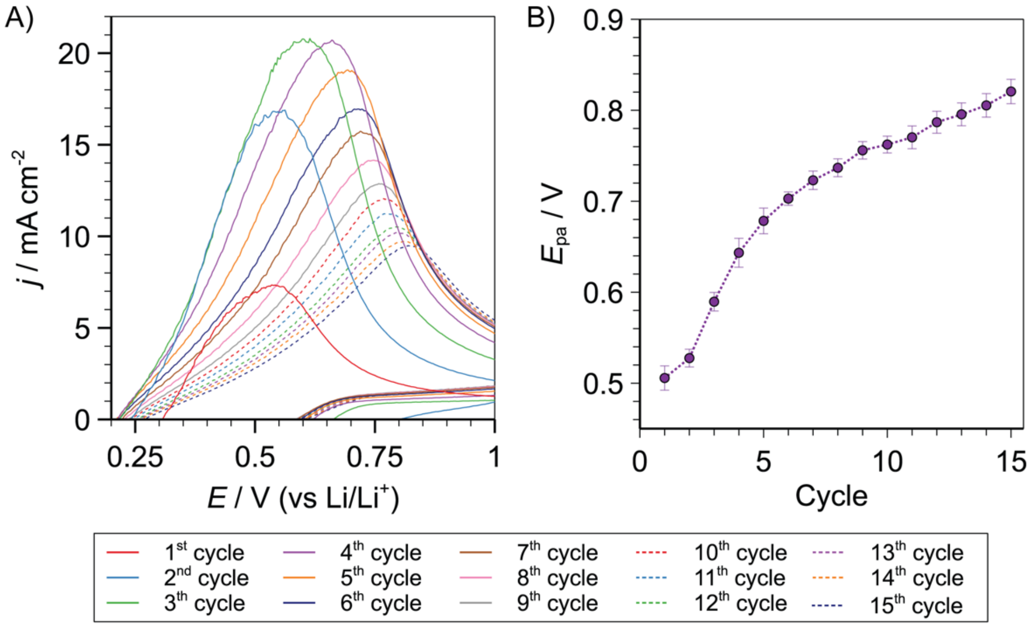

Figure S17. (A) Anodic component of representative CVs for 15 consecutive cycles recorded by SECCM in 1 M $LiPF_6$ in PC under high SOC (cut-off voltage: -0.13 V vs Li/Li^+). **(B)** Evolution of the anodic peak potential (E_{pa}) in function of charge/discharge cycle. E_{pa} values are the average of all the SECCM individual measurements (n=11).

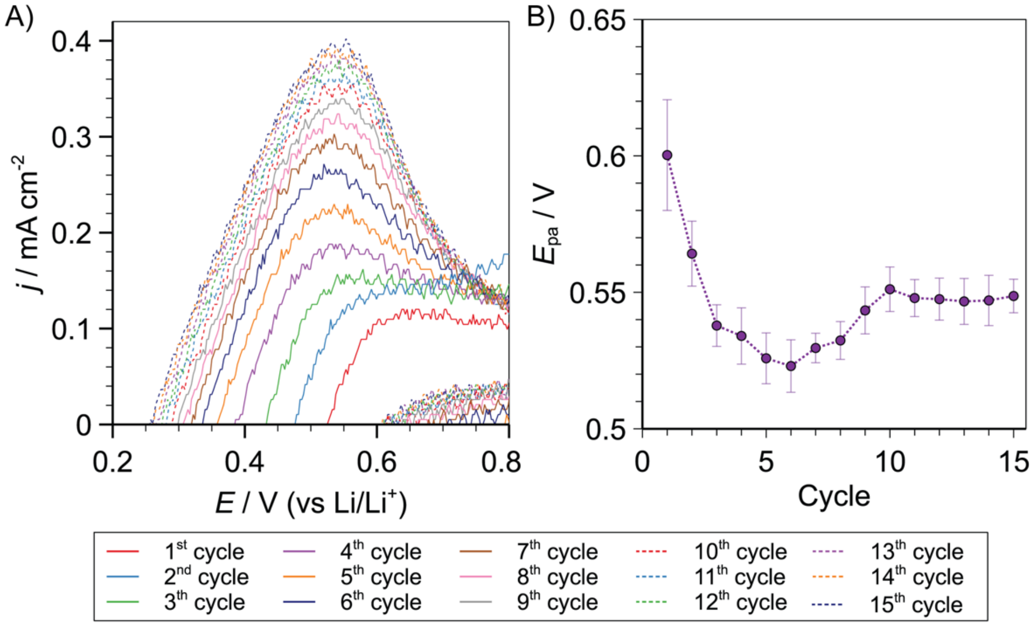

Figure S18. (A) Anodic component of representative CVs for 15 consecutive cycles recorded by SECCM in 1 M $LiPF_6$ in EC/EMC under low SOC (cut-off voltage: +0.05 V vs Li/Li^+). **(B)** Evolution of the anodic peak potential (E_{pa}) in function of charge/discharge cycle. E_{pa} values are the average of all the SECCM individual measurements (n=11).

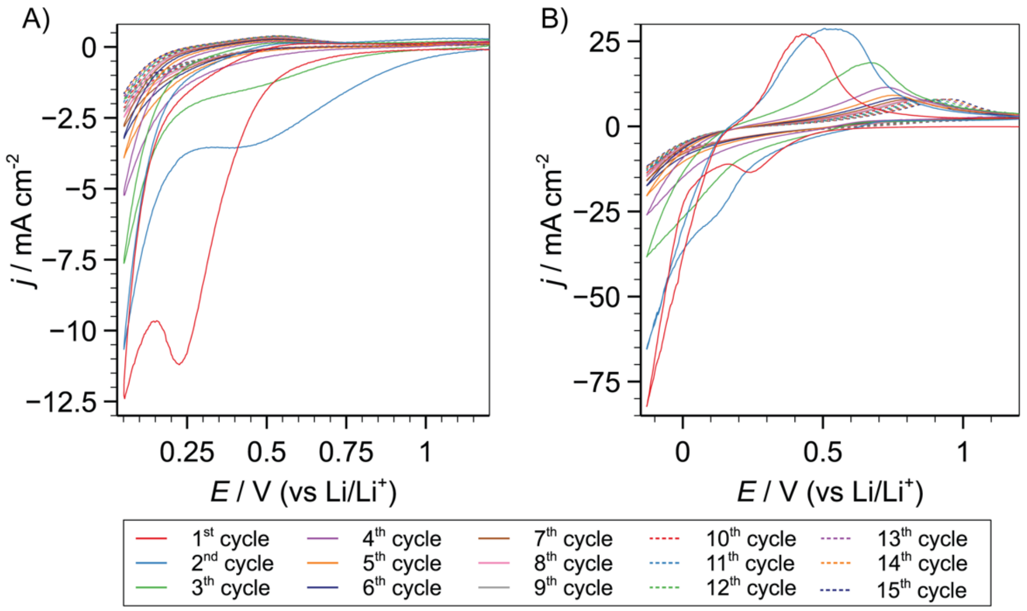

Figure S19. Representative CVs for 15 consecutive cycles recorded by SECCM in 1 M $LiPF_6$ in EC/EMC under low (A) and high (B) SOC (cut-off voltages of +0.05 and -0.13 V vs Li/Li^+, respectively).

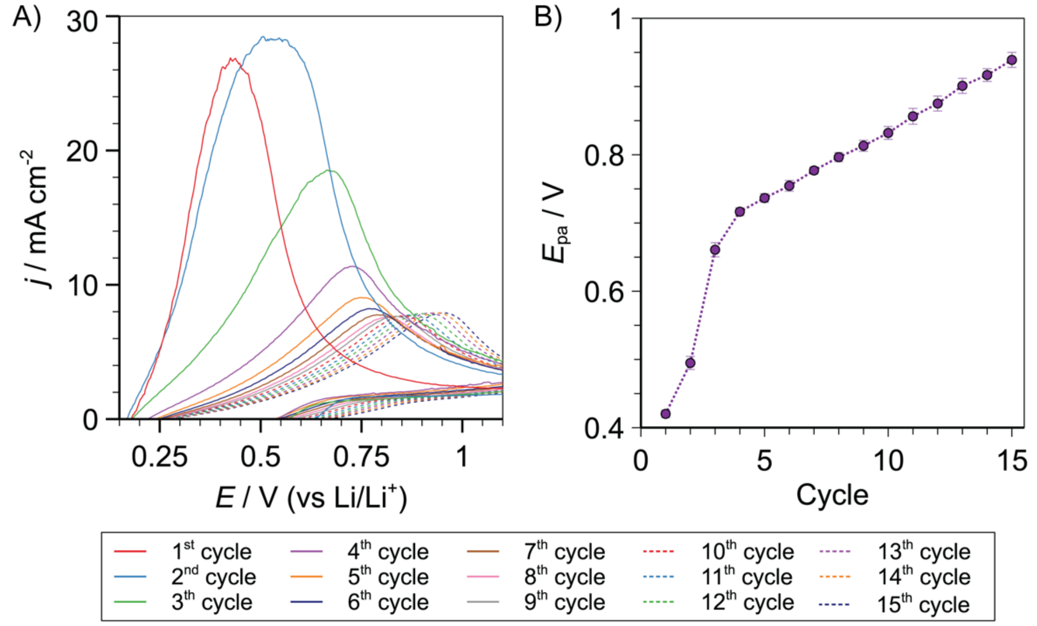

Figure S20. **(A)** Anodic component of representative CVs for 15 consecutive cycles recorded by SECCM in 1 M $LiPF_6$ in EC/EMC under high SOC (cut-off voltage: -0.13 V vs Li/Li^+). **(B)** Evolution of the anodic peak potential (E_{pa}) in function of charge/discharge cycle. E_{pa} values are the average of all the SECCM individual measurements (n=11).

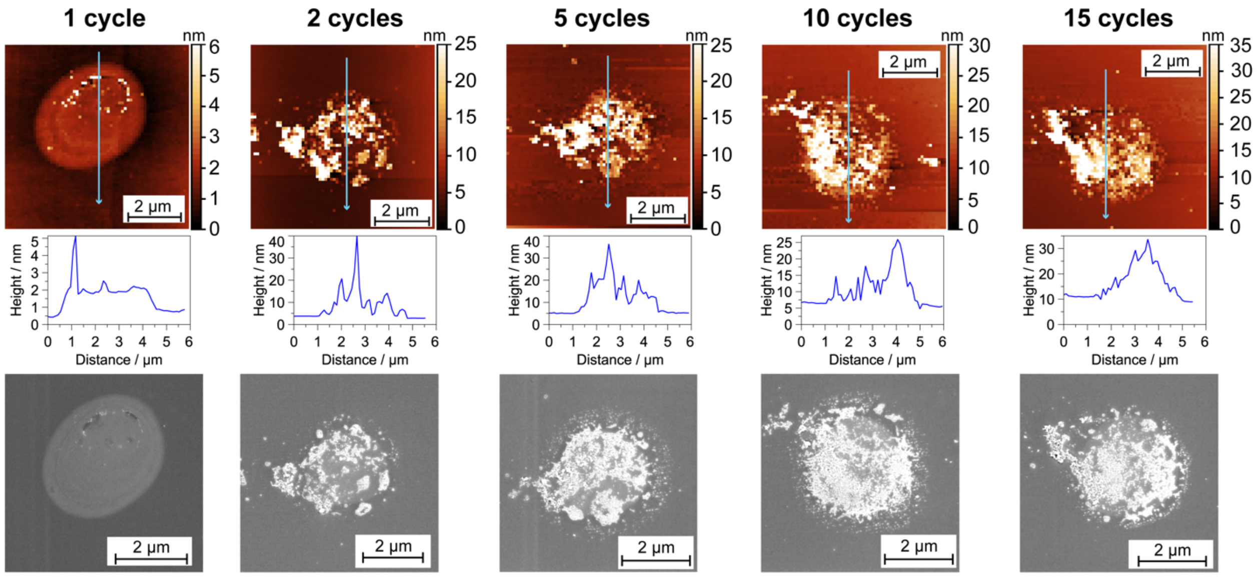

Figure S21. AFM topography images with corresponding height profiles and SEM images for the SEI layer prepared through SECCM for 1, 2, 5, 10 and 15 cycles in 1 M $LiPF_6$ in EC/EMC under low SOC (cut-off voltage: +0.05 V vs Li/Li^+). Arrows indicate the location and direction where the height profiles were extracted.

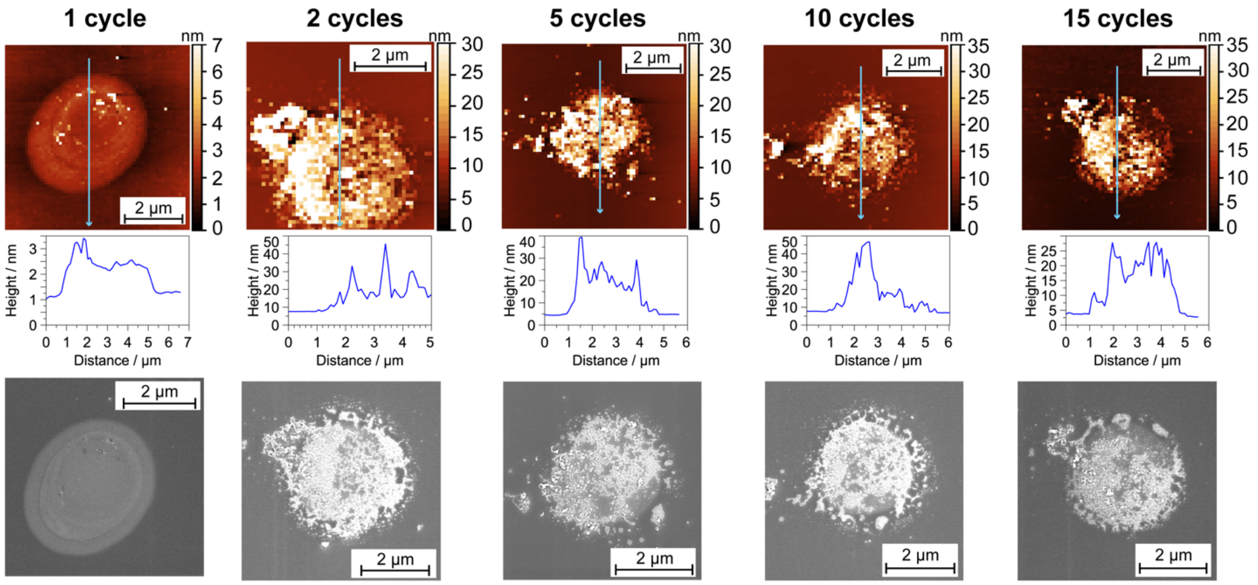

Figure S22. AFM topography images with corresponding height profiles and SEM images for the SEI layer prepared through SECCM for 1, 2, 5, 10 and 15 cycles in 1 M $LiPF_6$ in EC/EMC under high SOC (cut-off voltage: -0.13 V vs Li/Li^+). Arrows indicate the location and direction where the height profiles were extracted.

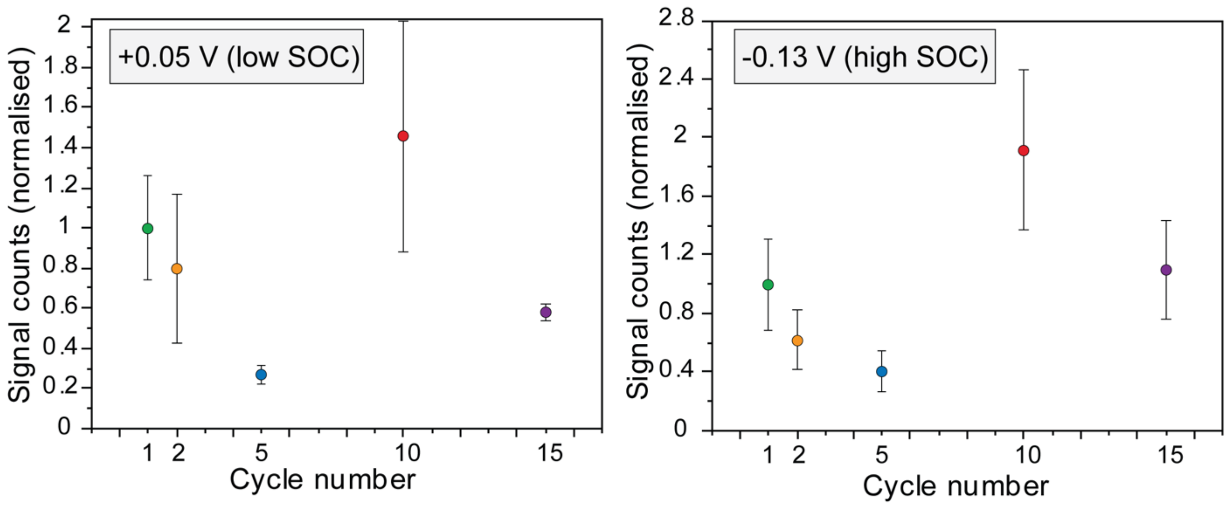

Figure S23. Averaged background emission intensity (normalised to the intensity of cycle 1) prior to baseline correction across all detected Raman bands in function of cycle number for the entire set of SECCM locations in 1 M $LiPF_6$ in EC/EMC at low SOC (cut-off voltage: +0.05 V vs Li/Li^+) and high SOC (cut-off voltage: -0.13 V vs Li/Li^+). Error bars represent the standard deviation at each wavelength of identified bands recorded for all different SECCM locations.

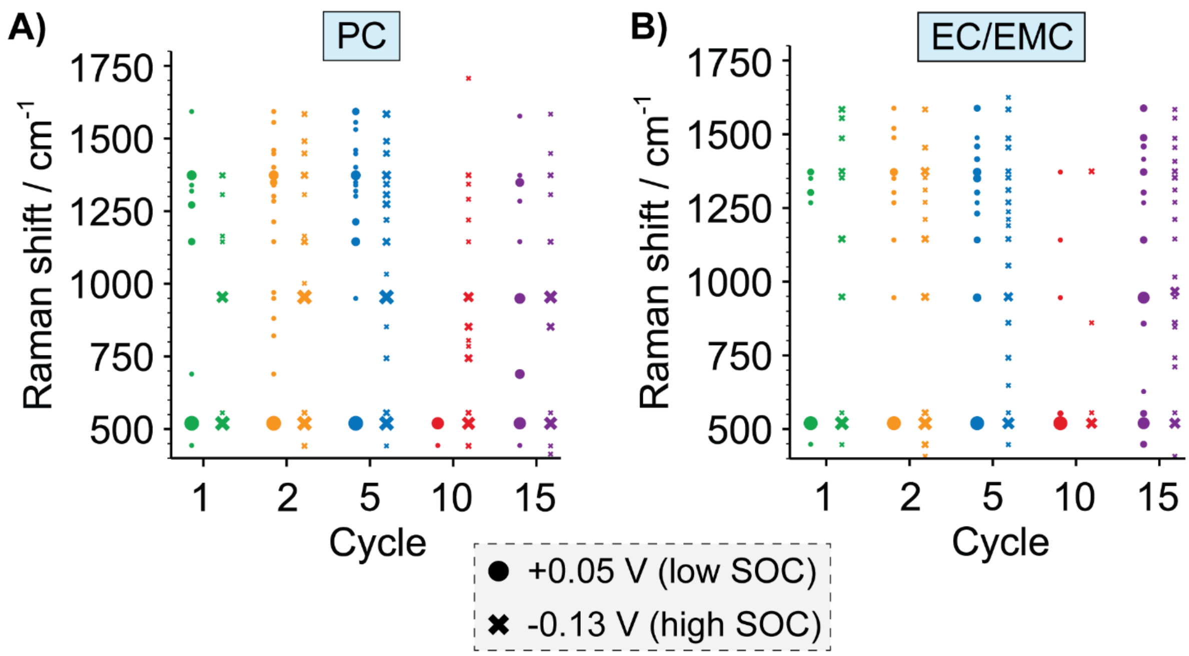

Figure S24. List of all Raman bands detected for the entire set of SECCM locations in function of cycle and SOC for 1 M $LiPF_6$ in PC **(A)** and EC/EMC **(B)**. Dot size represents the incidence number for a specific Raman band (note band at 520 cm^{-1} is from silicon wafer).

Table S1. State of the art on Raman studies for characterisation of SEI on Si electrodes for Li-ion batteries.

Reference	Method	Electrode	Electrolyte	Summary observation
Holzapfel et al (2006)[15]	First *in situ* Raman study	20% Nano-Si (ca. 20 nm) 80% graphite composite	1M $LiClO_4$, EC:DMC 1:1	The formation of first lithiated phases caused disappearance of the Si signal at 520 cm^{-1}. No SEI bands observed. Conventional Raman not sensitive enough.
Hy et al (2014)[16]	In situ SHINERS	Nano-Si (ca. 100 nm)	1M $LiPF_6$ EC:DEC 1:1 without and with 2 wt.% VC	Several weak bands, assignments for specific species challenging due to the similarities of the functional groups. Bands for Li_2CO_3 observed as well as slight fluorescent effect. Specific new band at 1000 cm^{-1} in vinylene carbonate (VC) electrolyte as key differentiation.
Nanda et al. (2019)[17]	TERS	Amorphous Si	1M $LiPF_6$ EC:DEC 1:1	Multiple peaks in 1450-1650 cm^{-1} region.<br><br>SEI composition gradually evolves with progressive cycling. Dominant SEI species at probed areas are LEDC and PEO-like oligomers for 1× a-Si, LEDCs for 5× a-Si, and carboxylate compounds for 20× a-Si.
Ha et al. (2020)[18]	SERS from nanostructure Cu mesh current collector	Amorphous Si	1.2M $LiPF_6$ EC:EMC 3:7	Broad peaks at 1430 and 1565 cm^{-1} tentatively assigned to νCOO^- Asym. and Sym. of lithium propanoate
This work (2022)	SHINERS combined with SECCM	Si (111) wafer	1 M $LiPF_6$ in PC and 1 M $LiPF_6$ in EC: EMC 1:1	Observe changing emission background as function of cycle number. Evidence of dynamic nature of SEI on Si.<br>Observe a series of variable bands and bands pertaining to PEO-type polymeric species.<br>Bands focused in 1330-1650 cm^{-1} region.

Table S2. Summary of main Raman bands and their assignments obtained from all the entire set of SECCM measurements.

Peak (cm^{-1})	Assignment	Ref.
300	Au-SiO_2 SHIN background	-
520	Si	[19]
839	$ROCO_2Li$ $\delta(OCO_2)$	[20]
941	LMC $\delta(OCO_2)_b$	[21]
941	PC	[21]
1138	PC	[21]
1145	PEO $(C\text{-}C/C\text{-}O\text{-}C)_{stretching}$	[21,22]
1212	PEO $(CH_2)_{torsion}$	[22]
1228	PEO $(CH_2)_{torsion}$	[21,22]
1260	PEO $(CH_2)_{asymmetric\ torsion}$	[22]
1281	PEO $(CH_2)_{asymmetric\ torsion}$	[21,22]
1300	$ROCO_2Li$ υ_s(C⎓O)	[20,22]
1341	$ROCO_2Li$ υ_s(C⎓O)	[20,22]
1367	Au-SiO_2 SHIN background	-
1445	δCH_2 (EC, EMC and PC) + LMC	[21]
1485	LMC	[21]
1550	Bridging + monodentate carboxylate	[17]
1580	Monodentate carboxylate RCOOLi υ_{as}(C⎓O)	[20,22]
1628	Monodentate carboxylate $ROCO_2Li$ $\upsilon_{as}(C{=}O)$	[20,22]
1687	$ROCO_2Li$ $\upsilon_{as}(C{=}O)$	[20,22]

Complex Raman spectra are measured in-line with expectations that the SEI is an evolving structure made up of a range of chemical species in differing environments. This complexity makes definitive assignments to individual species impractical. Previous studies have identified $ROCO_2Li$ and RCOOLi as products of PC decomposition[20,23] and modes here around 1300 cm^{-1} and 1500-1700 cm^{-1} could be reasonably assigned to these species. Evidence for PC being present at the surface is through the presence of known modes at ca. 940 and 1140 cm^{-1}.[21] Similarly experiments using EC/EMC are in-line with formation of complex mixtures containing species such as RCOOLi or $ROCO_2Li$ (ca. 1340 cm^{-1}, 1500-1600 cm^{-1}).[20,22,23] In some spectra bands pertaining directly to lithium methyl carbonate (LMC) can also be tentatively assigned. In all spectra the strong Raman mode at 520 cm^{-1} is due to the Si itself.

S3. REFERENCES

[1] H. Bülter, F. Peters, J. Schwenzel, G. Wittstock, *Angew. Chem. Int. Ed.* **2014**, *53*, 10531–10535.
[2] D. Martín-Yerga, M. Kang, P. R. Unwin, *ChemElectroChem* **2021**, *8*, 4240–4251.
[3] C.-H. Chen, L. Jacobse, K. McKelvey, S. C. S. Lai, M. T. M. Koper, P. R. Unwin, *Anal. Chem.* **2015**, *87*, 5782–5789.
[4] O. J. Wahab, M. Kang, P. R. Unwin, *Curr. Opin. Electrochem.* **2020**, *22*, 120–128.
[5] SciPy 1.0 Contributors, P. Virtanen, R. Gommers, T. E. Oliphant, M. Haberland, T. Reddy, D. Cournapeau, E. Burovski, P. Peterson, W. Weckesser, J. Bright, S. J. van der Walt, M. Brett, J. Wilson, K. J. Millman, N. Mayorov, A. R. J. Nelson, E. Jones, R. Kern, E. Larson, C. J. Carey, İ. Polat, Y. Feng, E. W. Moore, J. VanderPlas, D. Laxalde, J. Perktold, R. Cimrman, I. Henriksen, E. A. Quintero, C. R. Harris, A. M. Archibald, A. H. Ribeiro, F. Pedregosa, P. van Mulbregt, *Nat. Methods* **2020**, *17*, 261–272.
[6] J. Turkevich, *Gold Bull.* **1985**, *18*, 86–91.
[7] J. Turkevich, P. C. Stevenson, J. Hillier, *Discuss. Faraday Soc.* **1951**, *11*, 55.
[8] G. Frens, *Kolloid-Z. Z. Für Polym.* **1972**, *250*, 736–741.
[9] G. Frens, *Nat. Phys. Sci.* **1973**, *241*, 20–22.
[10] J. F. Li, Y. F. Huang, Y. Ding, Z. L. Yang, S. B. Li, X. S. Zhou, F. R. Fan, W. Zhang, Z. Y. Zhou, D. Y. Wu, B. Ren, Z. L. Wang, Z. Q. Tian, *Nature* **2010**, *464*, 392–395.
[11] W. Haiss, N. T. K. Thanh, J. Aveyard, D. G. Fernig, *Anal. Chem.* **2007**, *79*, 4215–4221.
[12] Ž. Krpetić, A. M. Davidson, M. Volk, R. Lévy, M. Brust, D. L. Cooper, *ACS Nano* **2013**, *7*, 8881–8890.
[13] A. M. Davidson, M. Brust, D. L. Cooper, M. Volk, *Anal. Chem.* **2017**, *89*, 6807–6814.
[14] T. A. Galloway, L. J. Hardwick, *J. Phys. Chem. Lett.* **2016**, *7*, 2119–2124.
[15] M. Holzapfel, H. Buqa, L. J. Hardwick, M. Hahn, A. Würsig, W. Scheifele, P. Novák, R. Kötz, C. Veit, F.-M. Petrat, *Electrochimica Acta* **2006**, *52*, 973–978.
[16] S. Hy, Felix, Y.-H. Chen, J. Liu, J. Rick, B.-J. Hwang, *J. Power Sources* **2014**, *256*, 324–328.
[17] J. Nanda, G. Yang, T. Hou, D. N. Voylov, X. Li, R. E. Ruther, M. Naguib, K. Persson, G. M. Veith, A. P. Sokolov, *Joule* **2019**, *3*, 2001–2019.
[18] Y. Ha, B. J. Tremolet de Villers, Z. Li, Y. Xu, P. Stradins, A. Zakutayev, A. Burrell, S.-D. Han, *J. Phys. Chem. Lett.* **2020**, *11*, 286–291.
[19] A. Krause, O. Tkacheva, A. Omar, U. Langklotz, L. Giebeler, S. Dörfler, F. Fauth, T. Mikolajick, W. M. Weber, *J. Electrochem. Soc.* **2019**, *166*, A5378–A5385.
[20] P. Verma, P. Maire, P. Novák, *Electrochimica Acta* **2010**, *55*, 6332–6341.
[21] A. Gajan, C. Lecourt, B. E. Torres Bautista, L. Fillaud, J. Demeaux, I. T. Lucas, *ACS Energy Lett.* **2021**, *6*, 1757–1763.
[22] L. Cabo-Fernandez, D. Bresser, F. Braga, S. Passerini, L. J. Hardwick, *Batter. Supercaps* **2019**, *2*, 168–177.
[23] Y. Chu, Y. Shen, F. Guo, X. Zhao, Q. Dong, Q. Zhang, W. Li, H. Chen, Z. Luo, L. Chen, *Electrochem. Energy Rev.* **2020**, *3*, 187–219.
